# Congenital Anomalies in Contaminated Sites: A Multisite Study in Italy

**DOI:** 10.3390/ijerph14030292

**Published:** 2017-03-10

**Authors:** Michele Santoro, Fabrizio Minichilli, Anna Pierini, Gianni Astolfi, Lucia Bisceglia, Pietro Carbone, Susanna Conti, Gabriella Dardanoni, Ivano Iavarone, Paolo Ricci, Gioacchino Scarano, Fabrizio Bianchi, RiscRipro_Sentieri Working Group

**Affiliations:** 1 Institute of Clinical Physiology, National Research Council, Unit of Environmental Epidemiology and Disease Registries, 56124 Pisa, Italy; fabrizio.minichilli@ifc.cnr.it (F.M.); apier@ifc.cnr.it (A.P.); fabriepi@ifc.cnr.it (F.B.); 2 Registro IMER, Dipartimento di Scienze Biomediche e Chirurgico Specialistiche dell’Università di Ferrara, 44100 Ferrara, Italy; asg@unife.it; 3 Agenzia Regionale Sanitaria della Puglia, 70100 Bari, Italy; l.bisceglia@arespuglia.it; 4 National Center for Rare Diseases, Istituto Superiore di Sanità, 00161 Rome, Italy; pietro.carbone@iss.it; 5 Unit of Statistics, Istituto Superiore di Sanità, 00161 Rome, Italy; susanna.conti@iss.it; 6 Osservatorio Epidemiologico Regionale, Assessorato Salute Regione Siciliana, 90145 Palermo, Italy; gabriella.dardanoni@regione.sicilia.it; 7 Department of Environment and Health, Istituto Superiore di Sanità, 00161 Rome, Italy; ivano.iavarone@iss.it; 8 Epidemiological Unit, NHS Mantua, 46100 Mantua, Italy; paolo.ricci@ats-valpadana.it; 9 Program Director Birth Defects Registry of Campania, UO Genetica Medica, Azienda Ospedaliera G.Rummo, 82100 Benevento, Italy; gioacchino.scarano@ao-rummo.it

**Keywords:** contaminated sites, congenital anomalies, epidemiological surveillance

## Abstract

The health impact on populations residing in industrially contaminated sites (CSs) is recognized as a public health concern especially in relation to more vulnerable population subgroups. The aim of this study was to estimate the risk of congenital anomalies (CAs) in Italian CSs. Thirteen CSs covered by regional CA registries were investigated in an ecological study. The observed/expected ratios (O/E) with 90% confidence intervals (CI) for the total and specific subgroups of CAs were calculated using the regional areas as references. For the CSs with waste landfills, petrochemicals, and refineries, pooled estimates were calculated. The total number of observed cases of CAs was 7085 out of 288,184 births (prevalence 245.8 per 10,000). For some CSs, excesses for several CA subgroups were observed, in particular for genital and heart defects. The excess of genital CAs observed in Gela (O/E 2.36; 90% CI 1.73–3.15) is consistent with findings from other studies. For CSs including petrochemical and landfills, the pooled risk estimates were 1.10 (90% CI 1.01–1.19) and 1.07 (90% CI 1.02–1.13), respectively. The results are useful in identifying priority areas for analytical investigations and in supporting the promotion of policies for the primary prevention of CAs. The use of short-latency effect indicators is recommended for the health surveillance of the populations residing in CSs.

## 1. Introduction

Human health impacts in contaminated sites (CSs) are of great concern worldwide. The European Environment Agency has estimated there to be approximately 2.5 million potentially contaminated sites, many of which are related to industrial activities [1,2]. Census activities regarding CSs were recently carried out [3,4], and monitoring is currently in progress in Europe [5,6]. Industrial activities can cause widespread contamination, and the inadequate environmental management of these sites can potentially affect human health [7,8]. WHO provided a definition of CSs from a public health perspective: “Areas hosting or having hosted human activities which have produced or might produce environmental contamination of soil, surface or groundwater, air, food-chain, resulting or being able to result in human health impacts” [7]. This refers mainly to industrial activities, including waste disposal and treatment.

There are several approaches for assessing the impacts on human health most of which use an epidemiological approach [9,10]. A first level of epidemiological investigation is aimed at defining the health profile of the population residing in the proximity to CSs. The SENTIERI (Epidemiological Study of Residents in Italian National Priority Contaminated Sites) project has adopted a useful approach for defining the health status of residents in contaminated sites [11]. The project defines a framework of the health profile of populations residing in Italian contaminated sites defined by ministerial decrees as remediation sites of national interest. Mortality, hospitalization, and cancer incidence of populations residing near CSs have been investigated by epidemiological studies [11,12,13,14]. The health impact on populations residing in CSs is a public health concern, especially in relation to the most vulnerable population subgroups. Infants and fetuses are more susceptible than adults to environmental contaminants [15]. Of the birth outcomes, congenital anomalies (CAs) represent an important topic that is investigated worldwide. Major CAs are estimated to be diagnosed in 2%–4% of births [16]. CAs are considered a major cause of fetal death, infant mortality and morbidity, and long-term disability [17,18]. CAs are diseases with a high impact on affected individuals, their families, and the community in terms of quality of life and healthcare service needs [19]. The etiology of most CAs is multifactorial. WHO estimated that 5% (range 1%–10%) of CAs are attributable to environmental causes [20] and the gene-environment interaction can contribute to the causation process [21].

Population-based registries of CAs are the main source of CA prevalence data [21]. CA registries are tools used for identifying genetic and teratogenic exposures, as well as evaluating and planning health care services and prevention policies [19]. CA registries are also effective tools for epidemiological assessments in polluted areas [22]. Research and monitoring focused on the potential environmental causes of CAs provide useful information aimed at generating hypotheses in order to identify teratogens. Following a feasibility assessment on the epidemiological study of adverse reproductive outcomes in small polluted areas, the Joint Action EUROCAT 2011–2013 reported that, “currently, links between reproductive health and contaminated sites are studied in a non-extensive and non-integrated way in Europe” [22]. Studying the impact of environmental causes on CAs should be based on an epidemiological approach and is a very complex field of investigation [16,21]. The association of CAs with industrially CSs has not been adequately investigated. A systematic evaluation of the epidemiological evidence reported the association between CAs and exposure to landfills, petrochemical plants, and/or refineries as being limited, and the association with other industrial sources as inadequate or not evaluable [23]. Few analytical epidemiological studies aimed at investigating selected CAs in industrial areas have been performed [24,25,26] and no evidence of association or only slight association in specific subgroups were detected. Limited to the exposure to air pollution, evidence of association has been reported in two reviews, particularly in relation to specific congenital heart defects [27,28]. A few epidemiological studies, including geographic studies, have investigated the association of CAs with exposure to waste disposals, but results are not consistent, and the evidence of the association is evaluated as limited [29,30,31,32,33,34,35].

The aim of our study was to estimate the risk of congenital anomalies in various contaminated sites in Italy. To our knowledge, this is the first multi-site study performed in Europe on the risk of CAs in different types of industrially contaminated areas.

## 2. Materials and Methods

Italian CSs have been defined by Ministerial decrees and identified for remediation following a documented contamination with a potential health impact. They are recognized as remediation sites of national interest because of the relevance of the pollution considering health, environmental, and social criteria. The contamination is the result of direct emissions, or indirect releases from waste disposals, controlled or uncontrolled. These areas are characterized by various types of industrial activities and have been classified as: chemicals, petrochemicals and refineries, steel plants, power plants, mines and/or quarries, harbor areas, asbestos or other mineral fibers, landfills, and incinerators [11,12,13,14,23]. CSs have different levels of environmental contamination and information on specific chemical contaminants is not available for all sites [36]. Legislative decrees provide information about the type of industrial activity; in the same CS, multiple types of current and past activities can be present. The decrees have also defined the CS boundaries, which are constituted by a municipality or by aggregates of municipalities [23].

In our study, 13 CSs covered by existing CA registries were considered. We used data collected by the following Italian population-based registries: the Congenital Malformations Registry of Mantua; IMER-Emilia Romagna Registry of Congenital Malformations; the Tuscany Registry of Congenital Defects; Birth Defects Registry of Campania; and the Congenital Malformations Registry of Sicily.

Data included cases with CAs among live births (up to 1 year of age), fetal deaths (with gestational age of 20 weeks or more), and terminations of pregnancy for fetal anomaly. For the CSs in Sicily and Apulia, since the CA registries were set up recently, a specific algorithm for the detection of live birth cases with CAs by hospital discharge records was used [37,38]. This algorithm filters the hospital discharge record data with ICD9 codes 740-759, applying a priori criteria in order to classify individual records into three groups: “certain CA”, “uncertain CA”, “no CA”. In our study, we used only those cases with CA classified as certain by the algorithm.

The study period ranged from 1992 to 2012, with some differences between registries depending on the availability of data (Table 1). The number of births by municipality of mother’s residence and year of birth were extracted from the National Institute of Statistics [39].

We analyzed the total CAs and seven subgroups of CAs, defined according to the classification used by EUROCAT [40]. Isolated minor CAs were excluded according to the EUROCAT definitions. Cases with multiple anomalies were considered as a single case in the definition of total CAs. The following subgroups of CAs with relative ICD10-BPA codes were analyzed: nervous system (Q00–Q07), congenital heart defects (Q20–Q26), oro-facial clefts (Q35–Q37), digestive system (Q38–Q45, Q790), genital (Q50–Q52, Q54–Q56), urinary system (Q60–Q64, Q794), and limb (Q65–Q74).

For each CS, the number of observed cases for each subgroup of CAs was compared to the number of expected cases. Expected cases were calculated using the prevalence in the total area covered by the corresponding registry. We calculated the observed/expected ratio (O/E) with a 90% confidence interval (90% CI). We used a 90% CI in order to reduce the critical use of the CI as a surrogate of the hypothesis test [41] and in line with the previous studies on cancer, mortality and hospitalization performed in the same study areas. For the CSs with petrochemicals and/or refineries, and waste landfills, we calculated a pooled estimate of the risk of the total and the most frequent CA subgroups. The pooled estimates were calculated using a random effects meta-analysis applied to the single estimates calculated for each CS.

Data analysis was performed using SAS version 9.2 (SAS Institute Inc., Cary, NC, USA) and STATA version 13 (StataCorp LP, College Station, TX, USA).

## 3. Results

The study area is represented by 13 CSs covered by regional registries of CAs. Table 1 reports the sources of environmental exposure included in each CS. The total number of resident cases with CAs during the study period was 7085 out of 288,184 births, thus accounting for a prevalence of 245.8 per 10,000. In the reference areas, 51,501 cases with CAs were registered during the same time period out of 2,368,031 total births, accounting for a prevalence of 217.48 per 10,000. The number of cases of total CAs was higher than the expected value in the following CSs: Laghi di Mantova (O/E 1.15; 90% CI 1.03–1.29), Livorno (O/E 1.33; 90% CI 1.25–1.41), Piombino (O/E 1.51; 90% CI 1.31–1.73), Manfredonia (O/E 1.36; 90% CI 1.22–1.51), and Taranto (O/E 1.10; 90% CI 1.02–1.18) (Table 2).

Considering the subgroup of CAs, several excesses were observed in some CSs:

- in Laghi di Mantova for congenital heart defects (CHD) (O/E 1.37; 90% CI 1.15–1.62), digestive system (O/E 1.80; 90% CI 1.16–2.67), and genital (O/E 1.54; 90% CI 1.07–2.14);
- in Livorno for CHD (O/E 1.56; 90% CI 1.42–1.72), limb (O/E 1.60; 90% CI 1.37–1.85), and genital (O/E 1.44; 90% CI 1.19–1.73);
- in Manfredonia for CHD (O/E 1.55; 90% CI 1.34–1.79), digestive system (O/E 1.91; 90% CI 1.30–2.70), and urinary system (O/E 1.62; 90% CI 1.14–2.25);
- in Gela for CHD (O/E 1.22; 90% CI 1.02–1.45), genital (O/E 2.36; 90% CI 1.73–3.15), and urinary system (O/E 3.21; 90% CI 2.55–3.99).

The CA subgroups for which the highest number of excesses were observed were genital (in 6 CSs) and CHD (in 5 CSs).

For CSs characterized by the presence of landfills, petrochemical, and refinery plants, Table 3 reports the pooled estimates of total CAs and selected CA subgroups. For the pool of seven CSs including petrochemical and/or refinery plants, the pooled risk estimate was 1.10 (90% CI 1.01–1.19) for total CAs, 1.15 (90% CI 1.00–1.30) for CHD, and 1.24 (90% CI 1.02–1.47) for genital. The pooled estimate in the 11 CSs containing a landfill was 1.07 (90% CI 1.02–1.13) for total CAs, and 1.08 (90% CI 1.00–1.16) for CHD.

## 4. Discussion

The health impact assessment of industrially polluted areas is important for improving scientific knowledge and public health decision-making. It is a very complex process due to the multiple and heterogeneous sources of pollution, the role of other non-environmental risk factors, and the multifactorial etiology of the diseases [7]. We compared the prevalence of CAs in populations residing in the neighborhood of polluted areas with larger reference areas. We analyzed data from the CA registries. The disease registries are considered to be the most accurate data source to provide a better definition of health outcomes also related to polluted areas [11]. The CA prevalence estimates are being highly variable across the different registries due to different diagnostic practices and methods of gathering and coding data [21]. In our study, the prevalence of CAs in each CS was compared with the prevalence in the region including the CS. Thus, we used the same data sources for the study area and the reference area, thus avoiding bias due to differences in registration activities.

In some study areas, we used hospital discharge data filtered by applying a specific algorithm [37]. In a sensitivity study aimed at testing and validating this algorithm, a positive predictive value of “certain cases” equal to 93.5% was observed [38]. The algorithm produces a valid estimation of CA cases, but limited to live birth cases. The terminations of pregnancy due to fetal anomaly are estimated in about 17% of the total cases [42]. We performed a sensitivity analysis excluding FD and TOPFA cases in the study areas where all the types of events were collected. The results were very similar and some slight differences were observed only for the subgroup of the nervous system. This result was expected, as the percentage of TOPFA varies between CA subgroups, with higher values for CAs of the nervous system and of the abdominal wall, in addition to chromosomal anomalies [43]. Thus, for the subgroup of nervous system, the results relating to areas where only live births cases were considered should be interpreted with caution.

For some CSs, a high risk of CAs emerged from the data analysis: in Laghi di Mantova, Livorno, Gela, and Manfredonia, excesses for several subgroups of CAs were observed. In some of these areas, the occurrence of CAs had been investigated in previous epidemiological studies. A study on CAs performed in the CS of Laghi di Mantova from 2002 to 2006 detected an overall risk of 1.47 (95% CI 0.77–1.82), using the surrounding municipalities as reference area. In the CS of Gela, excesses of genital and urinary CAs were detected by two epidemiological studies [44,45]; in particular, the rates of hypospadias (56.7 and 46.7 per 10,000 respectively) were more than double compared to Italian and European references. More recently, an even higher prevalence of hypospadias (88.5 per 10,000) a 3.5-fold excess compared to the reference, was detected using data from the Congenital Malformations Registry of Sicily [46].

We also estimated risks considering the pool of CSs including landfills, petrochemical, and/or refineries. In order to limit the variability among the single estimates, we used a meta-analytical approach. Pooled risk estimates considering the CSs with petrochemical and/or refineries showed excesses for total, heart, and genital CAs. Since many CSs contain multiple sources of exposure other than those selected, the pooled estimates should be interpreted with caution. For the pool of eleven CSs including waste landfills, the meta-analytical estimate of risk showed excesses of total CAs and CHD.

The two CSs in Campania, namely Area Litorale Vesuviano and Litorale Domizio Flegreo e Agro Aversano, are large areas characterized by the widespread presence of heterogeneous waste disposal sites (controlled landfills, illegal dumps, illegal and uncontrolled waste disposal sites with hazardous waste). Epidemiological studies at municipality level performed in these areas have detected excesses for various CAs [47,48,49]. In our study, no excesses of CAs were observed in the two CSs. The wide geographical and demographic dimension of these areas, the heterogeneity of the sources of exposure, and the limitation due to the ecological design, need to be considered in the interpretation of the results.

We used an approach previously adopted for mortality, hospitalization, and cancer incidence studies in Italian CSs [11,12,13,14]. The methodology used in these studies is defined by WHO as a first level investigation at the population level within the wider framework of a funnel approach [7]. The ecological approach at the minimum aggregation level of the municipality determines the known limitations of this kind of design, mainly the ecological fallacy. Despite the limitations, the ecological design is consistent with the aim of describing the health profile of the population in industrially contaminated areas through a gradual investigation [7], as well as providing preliminary information, which is useful for the evolution of the research, especially when prior knowledge and evidence is limited [50]. Ecological studies also play a major role in the investigation of associations of public health importance [51]. The ecological design did not enable us to consider possible individual confounders related to maternal and paternal risk factors. Population living in the neighborhood of the CSs have tendentially a more disadvantaged socio-economic condition. We did not provide risk estimates adjusted for the socio-economic deprivation index at the municipality level due to the limitation of representing the socio-economic status for municipalities with more than 10,000 inhabitants [52]. The unavailability of quantitative assessments of population exposure is a limitation shared by other studies in the SENTIERI project [14]. A more accurate characterization of the exposure is needed in order to better fit the epidemiological investigation in the CSs.

As CAs are rare events, CAs were grouped in order to ensure an adequate statistical power. This was to hide a potential association with a specific anomaly, especially for subgroups with an etiological and pathogenetic heterogeneity [16]. Our study detected excesses in particular for the heart and genital subgroups. Further studies, with a sufficient number of cases, are needed to investigate the association of selected subgroups or single anomalies.

The present study aimed to improve the description of the health profile of residing populations in CSs previously investigated through other health outcomes, most of which were long-term. The results do not support the potentially causal role of the risk of CAs in terms of pollution in contaminated areas; however, the detected risks suggest etiological hypotheses and support further studies.

Finally, the study consolidates the recommendations that emerged as part of the European Joint Action EUROCAT, which identified CA registries as a primary information source for epidemiological surveillance in contaminated areas through a linkage with environmental pollution data [22].

## 5. Conclusions

The results of this multi-site study identified areas with excesses of different subgroups of CAs. The results are useful for identifying priority areas for analytical investigations, aimed at etiological research and intervention studies. The study supports planning for primary prevention of CAs, thus mainly the remediation of contaminated areas. The results on CAs complete the epidemiological framework in areas where different environmental stressors produce different health effects. A multi-outcome approach (mortality, cancer incidence, hospitalization, and congenital anomalies) contributes to understanding the role of environmental factors in the health risk profile of populations residing in contaminated areas. Fetuses and pregnant women comprise vulnerable subgroups sensitive to environmental insults and require specific surveillance. Our findings reinforce the recommendation of using short-latency effect indicators, such as the prevalence of CAs, for the health surveillance of populations residing in contaminated areas. It would be desirable to expand the surveillance of CAs to a wider international context in populations living in proximity to hazardous areas.

## Figures and Tables

**Table 1:** Contaminated sites, sources of environmental exposure, registry of congenital anomalies, type of cases, study period, and number of births.

Contaminated Site	Environmental Exposure	Registry of Congenital Anomalies	Type of Cases	Study Period	*n*. Births
Laghi di Mantova	C, P&R, H, L	Mantova province	LB + FD + TOPFA	2002–2011	4872
Fidenza	C, L	Emilia Romagna region	LB + FD + TOPFA	1995–2011	5816
Sassuolo-Scandiano	C	Emilia Romagna region	LB + FD + TOPFA	1995–2011	19,323
Livorno	P&R, H	Tuscany region	LB + FD + TOPFA	1992–2011	27,511
Massa Carrara	C, P&R, S, H, L, A, I	Tuscany region	LB + FD + TOPFA	1992–2011	20,752
Piombino	C, S, E, H, L	Tuscany region	LB + FD + TOPFA	1992–2011	4660
Area Litorale Vesuviano	A, L	Campania region	LB + FD + TOPFA	2004–2010	34,452
Litorale Domizio Flegreo e Agro Aversano	L	Campania region	LB + FD + TOPFA	2004–2010	117,364
Gela	C, P&R, L	Sicily region	LB	2006–2010	4279
Priolo	C, P&R, H, L, A	Sicily region	LB	2006–2010	8592
Brindisi	C, P&R, E, H, L	Apulia region	LB	2001–2012	9925
Manfredonia	C, L	Apulia region	LB	2001–2012	8364
Taranto	P&R, S, H, L	Apulia region	LB	2001–2012	22,274

C = production of chemical substance/s; P&R = petrochemical plant and/or refinery; S = steel industry; E = electric power plant; H = harbor area; A = asbestos/other mineral fibres; L = landfill; I = incinerator; LB = live births; FD = fetal deaths; TOPFA = termination of pregnancy for fetal anomaly.

**Table 2:** Number of cases (N), observed/expected ratio (O/E) with 90% confidence interval (90% CI) by contaminated site and subgroup of congenital anomalies.

Contaminated Site	Total Congenital Anomalies		Nervous System		Congenital Heart Defects		Oro-Facial Clefts		Digestive System		Limb		Genital		Urinary System	
	N	O/E (90% CI)	N	O/E (90% CI)	N	O/E (90% CI)	N	O/E (90% CI)	N	O/E (90% CI)	N	O/E (90% CI)	N	O/E (90% CI)	N	O/E (90% CI)
Laghi di Mantova	221	1.15 (1.03–1.29)	23	1.31 (0.90–1.86)	99	1.37 (1.15–1.62)	7	0.98 (0.46–1.83)	18	1.80 (1.16–2.67)	25	0.86 (0.60–1.20)	25	1.54 (1.07–2.14)	24	1.00 (0.69–1.40)
Fidenza	97	1.00 (0.84–1.18)	5	0.69 (0.27–1.45)	26	0.91 (0.64–1.26)	5	0.94 (0.37–1.97)	10	1.35 (0.73–2.29)	20	1.32 (0.88–1.92)	21	2.27 (1.52–3.27)	4	0.33 (0.11–0.76)
Sassuolo-Scandiano	280	0.87 (0.78–0.96)	12	0.50 (0.29–0.81)	85	0.89 (0.74–1.07)	21	1.19 (0.80–1.71)	32	1.30 (0.95–1.74)	40	0.79 (0.60–1.03)	32	1.04 (0.76–1.40)	35	0.87 (0.64–1.15)
Livorno	745	1.33 (1.25–1.41)	43	1.13 (0.87–1.46)	303	1.56 (1.42–1.72)	25	1.02 (0.71–1.43)	34	1.01 (0.74–1.34)	126	1.60 (1.37–1.85)	82	1.44 (1.19–1.73)	66	1.04 (0.84–1.27)
Massa Carrara	438	1.04 (0.96–1.12)	24	0.84 (0.58–1.18)	147	1.00 (0.87–1.15)	17	0.92 (0.59–1.38)	33	1.30 (0.95–1.73)	44	0.74 (0.57–0.95)	40	0.93 (0.70–1.21)	53	1.10 (0.87–1.39)
Piombino	143	1.51 (1.31–1.73)	5	0.78 (0.31–1.63)	47	1.43 (1.11–1.82)	8	1.93 (0.96–3.49)	6	1.05 (0.46–2.07)	19	1.42 (0.93–2.09)	17	1.76 (1.12–2.65)	16	1.48 (0.93–2.25)
Area Litorale Vesuviano	821	0.94 (0.88–0.99)	94	0.99 (0.83–1.18)	313	0.94 (0.85–1.03)	35	0.70 (0.52–0.93)	56	0.93 (0.74–1.17)	116	1.10 (0.94–1.28)	103	0.90 (0.76–1.06)	93	1.06 (0.88–1.26)
Litorale Domizio Flegreo e Agro Aversano	2975	1.00 (0.97–1.03)	317	0.98 (0.89–1.08)	1,169	1.03 (0.98–1.08)	170	0.99 (0.87–1.13)	218	1.07 (0.95–1.19)	359	1.00 (0.91–1.09)	378	0.97 (0.89–1.05)	297	0.99 (0.90–1.09)
Gela	139	1.14 (0.99–1.31)	2	0.34 (0.06–1.06)	93	1.22 (1.02–1.45)	5	1.37 (0.54–2.88)	7	1.01 (0.47–1.90)	14	1.10 (0.67–1.72)	33	2.36 (1.73–3.15)	59	3.21 (2.55–3.99)
Priolo	242	0.99 (0.89–1.10)	15	1.27 (0.78–1.95)	140	0.91 (0.79–1.05)	3	0.41 (0.11–1.06)	23	1.65 (1.13–2.34)	32	1.26 (0.91–1.69)	21	1.54 (1.03–2.22)	13	0.69 (0.41–1.09)
Brindisi	206	0.96 (0.85–1.07)	11	1.02 (0.57–1.70)	105	1.03 (0.87–1.21)	6	0.48 (0.21–0.94)	11	0.77 (0.43–1.27)	19	0.72 (0.47–1.05)	32	0.90 (0.65–1.20)	25	1.32 (0.92–1.84)
Manfredonia	247	1.36 (1.22–1.51)	6	0.66 (0.29–1.31)	133	1.55 (1.34–1.79)	8	0.76 (0.38–1.37)	23	1.91 (1.30–2.70)	30	1.34 (0.97–1.82)	30	1.00 (0.72–1.35)	26	1.62 (1.14–2.25)
Taranto	531	1.10 (1.02–1.18)	33	1.37 (1.00–1.83)	236	1.03 (0.92–1.15)	37	1.32 (0.98–1.73)	37	1.15 (0.86–1.52)	87	1.46 (1.21–1.75)	79	0.99 (0.81–1.19)	49	1.15 (0.89–1.46)

**Table 3:** Pooled estimate (PE) with 90% confidence interval (90% CI) by source of exposure and subgroup of congenital anomalies.

Source of Exposure	Total Congenital Anomalies	Congenital Heart Defects	Genital	Limb	Urinary System	Nervous System
	PE (90% CI)	PE (90% CI)	PE (90% CI)	PE (90% CI)	PE (90% CI)	PE (90% CI)
Petrochemical plant and/or Refinery (7 CSs)	1.10 (1.01–1.19)	1.15 (1.00–1.30)	1.24 (1.02–1.47)	1.10 (0.85–1.36)	1.13 (0.99–1.28)	1.04 (0.83–1.24)
Landfill (11 CSs)	1.07 (1.02–1.13)	1.08 (1.00–1.16)	1.09 (0.96–1.23)	1.06 (0.94–1.18)	1.13 (0.94–1.32)	0.97 (0.87–1.06)

CS = contaminated site.
